# Mixed neuroendocrine–non-neuroendocrine neoplasm of the bile duct with long-term prognosis after neoadjuvant chemotherapy

**DOI:** 10.1007/s12328-024-01982-3

**Published:** 2024-05-24

**Authors:** Shinya Nakamura, Masahiro Serikawa, Yasutaka Ishii, Yumiko Tatsukawa, Juri Ikemoto, Sayaka Miyamoto, Kenichiro Uemura, Shinya Takahashi, Koji Arihiro, Shiro Oka

**Affiliations:** 1 Department of Gastroenterology, Graduate School of Biomedical and Health Sciences, Hiroshima University, 1-2-3 Kasumi Minami-Ku, Hiroshima, 734-8551 Japan; 2 Department of Gastroenterology, Hiroshima Prefectural Hospital, Hiroshima, Japan; 3 Department of Surgery, Graduate School of Biomedical and Health Science, Hiroshima University, Hiroshima, Japan; 4 Department of Pathology, Hiroshima University Hospital, Hiroshima, Japan

**Keywords:** Mixed neuroendocrine non-neuroendocrine neoplasm, Biliary tract, Neoadjuvant chemotherapy

## Abstract

A 74-year-old man with obstructive jaundice presented with a thickened distal bile duct wall. A transpapillary forceps biopsy revealed an adenocarcinoma; however, because the tumor image was different from that of a typical cholangiocarcinoma, endoscopic ultrasound-guided fine-needle aspiration was performed on the tumor and enlarged lymph nodes. The tumor cells were positive for synaptophysin and CD56 with a Ki67 labeling index of 95%, and he was diagnosed with small cell neuroendocrine carcinoma. We diagnosed a bile duct tumor with neuroendocrine carcinoma component with lymph node metastasis. Preoperative chemotherapy for neuroendocrine carcinoma was administered because R0 resection was difficult and the risk of postoperative recurrence was high. Three courses of chemotherapy with carboplatin and etoposide resulted in marked tumor shrinkage, and radical resection was performed 3 months after diagnosis. Postoperative pathology revealed adenocarcinoma in the mucosal epithelium and small cell neuroendocrine carcinoma in the submucosa, most of which resolved with chemotherapy. Carboplatin and etoposide were resumed as adjuvant chemotherapy, and 67 months of recurrence-free survival were achieved after surgery.

## Introduction

Neuroendocrine neoplasms (NEN) are derived from neuroendocrine cells and can occur in a variety of organs, although those primary to the biliary tract are rare [1–3]. Mixed neuroendocrine non-neuroendocrine neoplasms (MiNEN) are defined as mixed neoplasms with both neuroendocrine and non-neuroendocrine components, with either component representing at least 30% [1, 2]. A diagnosis of MiNEN of the biliary tract is difficult, and its prognosis is extremely poor. Therefore, the optimal treatment remains unknown [4]. Here, we report a case of primary MiNEN of the bile duct treated with chemotherapy for neuroendocrine carcinoma (NEC), followed by radical resection and postoperative adjuvant chemotherapy, which resulted in long-term recurrence-free survival.

## Case report

**Fig. 1:**
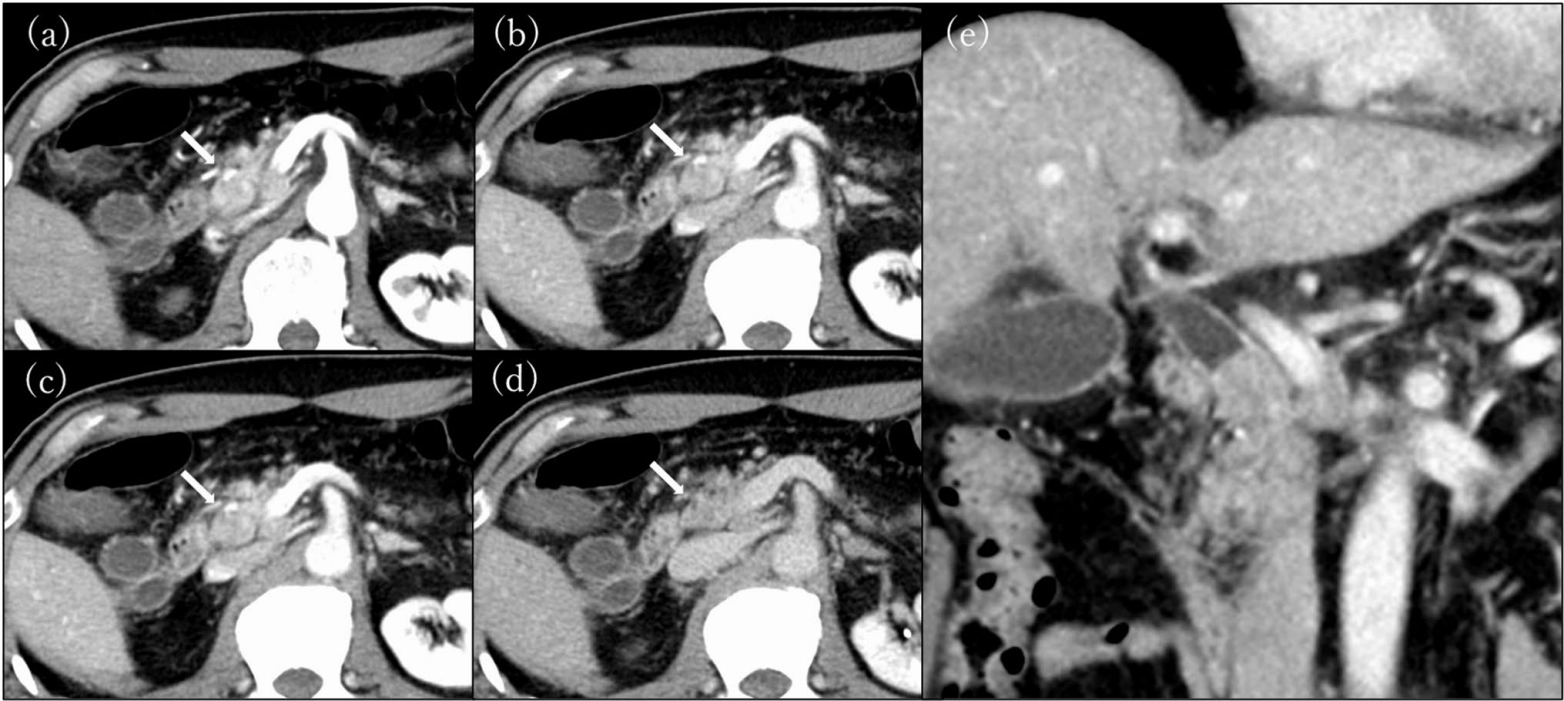
Contrast-enhanced computed tomography shows a tumor measuring 20 mm in the distal bile duct. The lesion is contrasted at the margins in the early phase and stains internally in the later phase **a** arterial phase; **b** pancreatic parenchymal phase; **c** portal phase; and **d** equilibrium phase. The biliary tract upstream of the lesion is dilated **(e)**

A 74-year-old man undergoing treatment for benign prostatic hyperplasia and glaucoma presented to our hospital with anorexia and epicardial pain that had persisted for 1 month. Blood test results were as follows: leucocyte count 4270 cells/μL, hemoglobin 14.2 g/dL, platelet count 24.0 × 10^4^ cells/μL, aspartate aminotransferase 285 IU/L, alanine aminotransferase 638 IU/L, total bilirubin 5.7 mg/dL, direct bilirubin 3.7 mg/dL, albumin 3.2 g/dL, C-reactive protein 0.02 mg/dL, carcinoembryonic antigen (CEA) 6.1 ng/mL, and carbohydrate antigen 19–9 (CA19-9) 30 U/mL. Abdominal ultrasonography revealed dilation of the common bile duct and the intrahepatic ducts. Contrast-enhanced computed tomography (CECT) revealed a 20-mm-sized nodular lesion in the distal bile duct with upstream bile duct dilatation. The lesion was contrasted at the margins in the early phase and stained internally in the later phases. Enlarged lymph nodes were observed near the pancreatic head. However, no abnormalities were observed in the liver, lungs, or bones (Fig. 1).

**Fig. 2:**
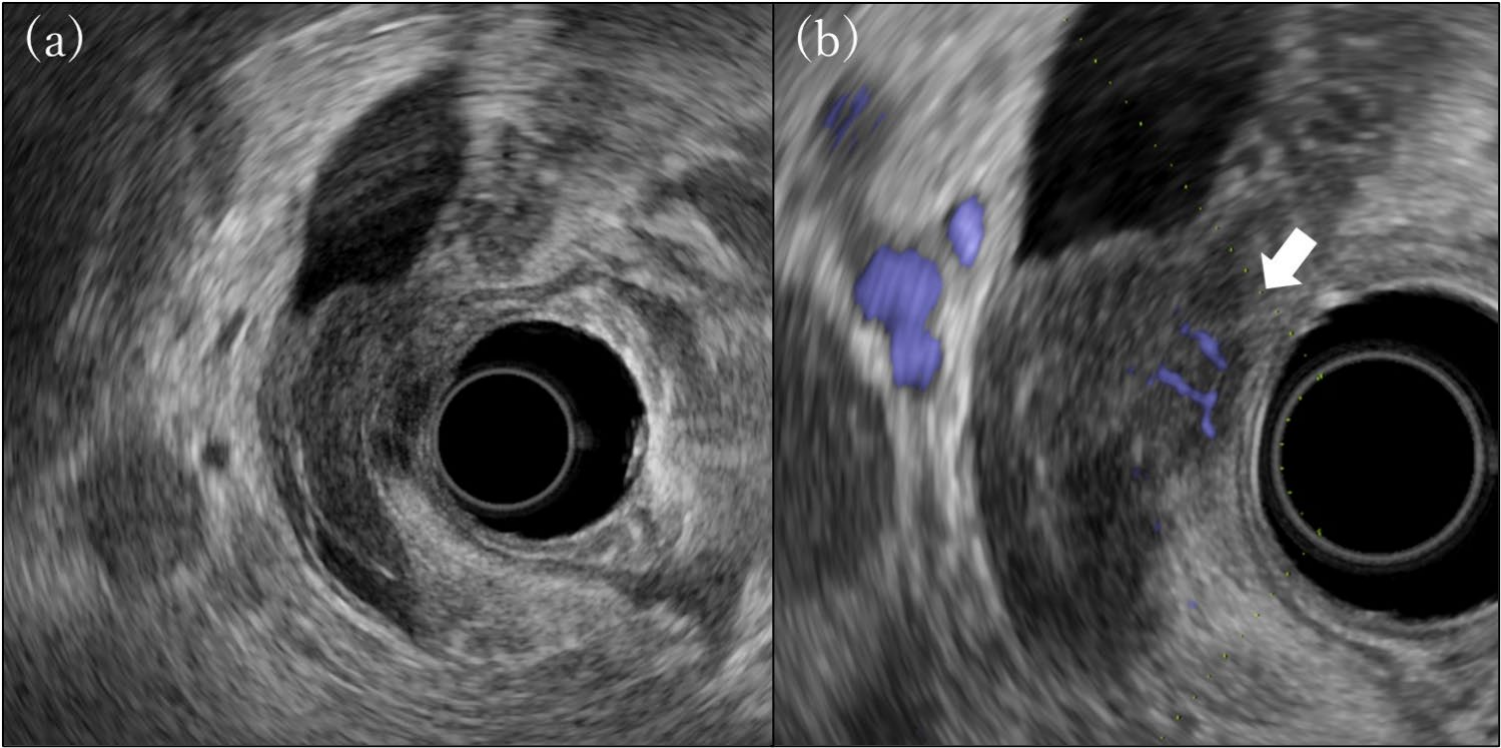
Endoscopic ultrasonography reveals wall thickening with a smooth mucosal side, but the outer high-echoic layer is partially disrupted **(a)**. An abundant blood flow signal is seen in the outer irregular region **(b)** (arrow)

Endoscopic ultrasonography (EUS) showed a homogeneous hypoechoic wall thickening in the distal bile duct. The mucosal side of the thickened wall was smooth. However, the outer hyperechoic layer was partially disrupted and extended into an irregularly contoured hypoechoic area. Numerous blood flow signals were observed in this area (Fig. 2).

Fluorodeoxyglucose positron emission tomography (FDG-PET) revealed FDG accumulation in the distal bile duct and lymph nodes (SUVmax 6.1 and 5.6, respectively).

Endoscopic retrograde cholangiopancreatography (ERCP) revealed a stricture with a smooth surface in the distal bile duct, and a transpapillary forceps biopsy of the stricture revealed an adenocarcinoma (ADC).

**Fig. 3:**
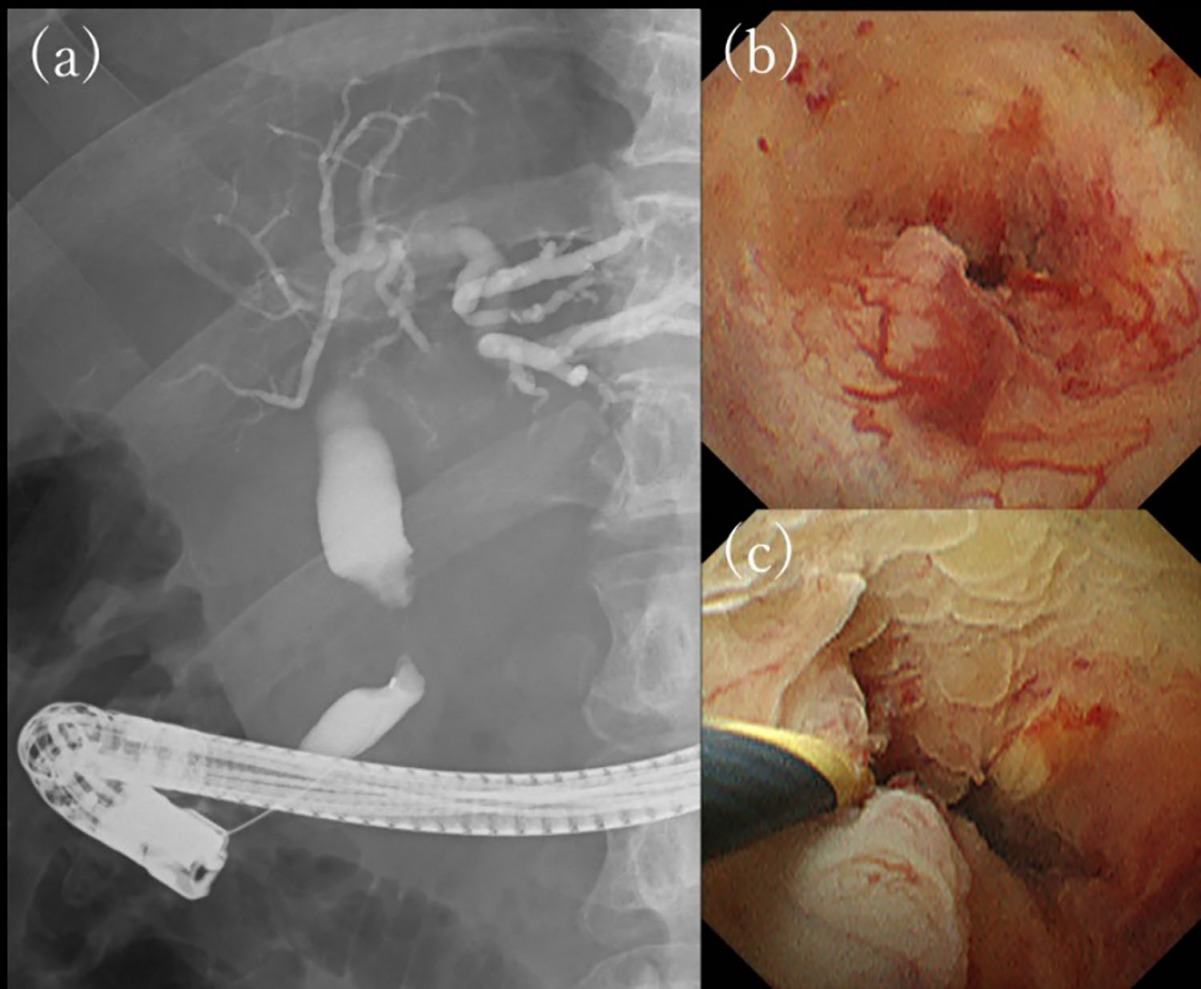
Endoscopic retrograde cholangiography reveals a stricture of the distal bile duct **(a)**. Peroral cholangioscopy reveals a submucosal tumor-like elevation in the lesion with dilated vessels **(b, c)**

Peroral cholangioscopy (POCS) was also performed. POCS revealed papillary, easily hemorrhagic mucosa, and dilated vessels, but these findings were localized to a part of the stricture (Fig. 3).

**Fig. 4:**
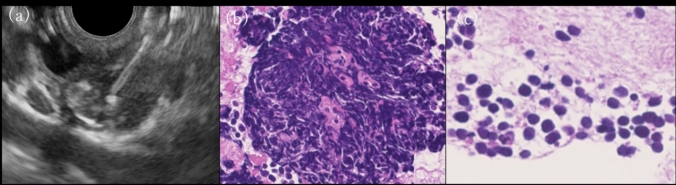
Endoscopic ultrasound-guided fine needle aspiration is performed on the tumor and nearby lymph nodes **(a)**. Small tumor cells with high nucleocytoplasmic ratios are seen in both lesions. **(b, c)**

Endoscopic ultrasound-guided fine-needle aspiration (EUS-FNA) was performed on the bile duct lesion and enlarged lymph nodes because of the possibility of a specific bile duct tumor. Small tumor cells with a high nucleocytoplasmic ratio that proliferated at a high rate, resulting in high cell density, were detected in both the bile duct and lymph nodes. The tumor cells were positive for synaptophysin and CD56 with a Ki67 labeling index of 95% and were diagnosed as small cell neuroendocrine carcinoma (SCNEC) (Fig. 4).

**Fig. 5:**
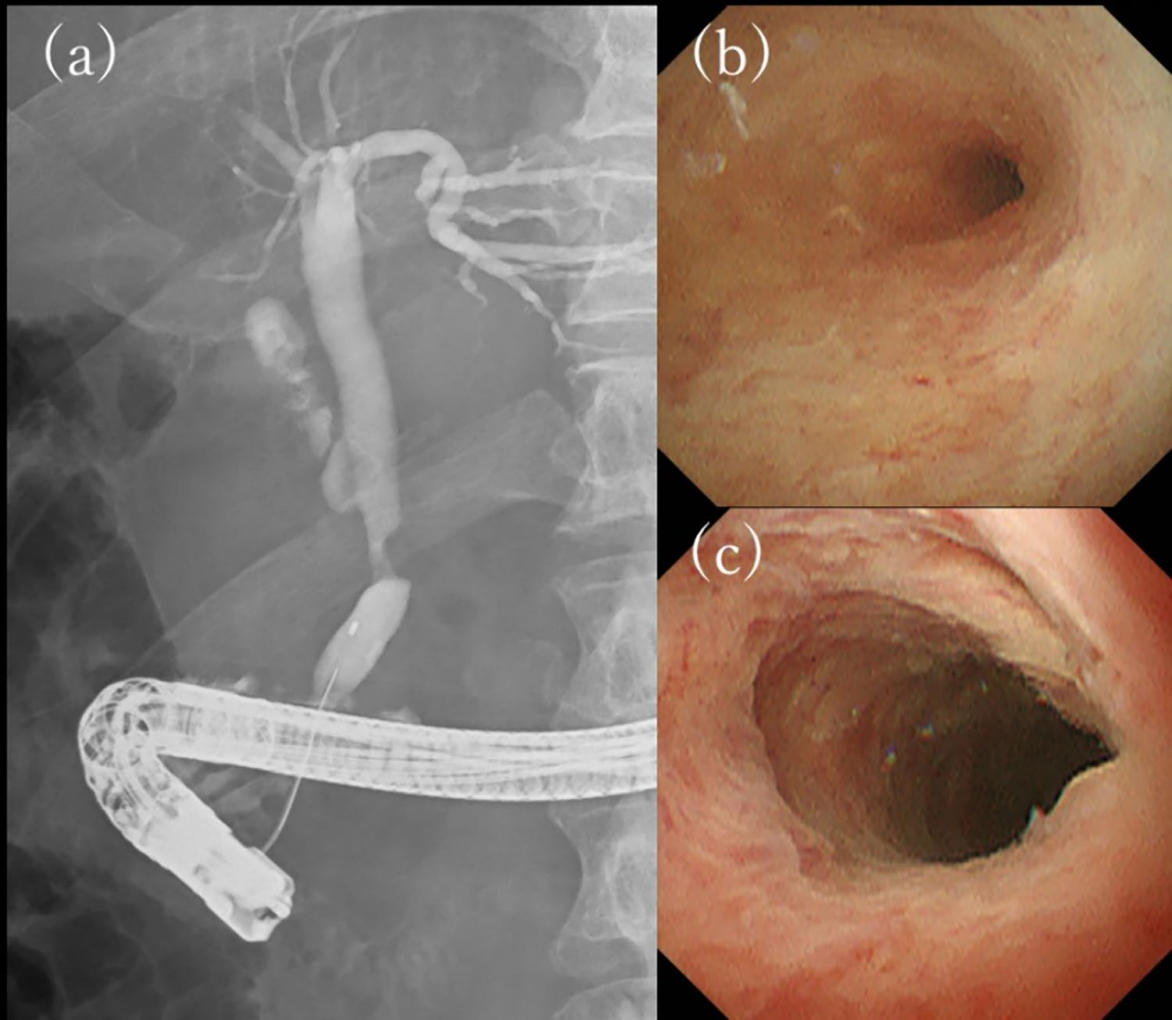
The tumor has markedly shrunken with chemotherapy **(a, b)**. The stricture has improved, revealing a whitish, scarred mucosa **(c, d, e)**

Pathological findings by EUS-FNA suggested a bile duct tumor with SCNEC and lymph node metastasis of the same component. Therefore, we diagnosed bile duct NEC or MiNEN, clinical stage IIB (T2N1M0, AJCC/UICC 8th edition). After discussing the treatment plan with the surgeon, it was determined that R0 surgery was unlikely and the risk of recurrence after resection was high because the tumor had invaded beyond the bile duct wall and was accompanied by lymph node metastasis of the NEC. Therefore, we decided to introduce chemotherapy with sufficient informed consent and started combination therapy with carboplatin (CBDCA) and etoposide (ETP) for the NEC. After three courses of treatment, CECT showed marked shrinkage of the tumor and lymph nodes, FDG-PET showed reduced FDG accumulation in the primary tumor, and lymph node metastases had decreased. ERCP and POCS were performed again to evaluate the tumor extent. Cholangiography revealed improvement of the stricture, POCS revealed that the mucosal surface was mostly white and scarred, and the submucosal prominence was reduced (Fig. 5).

**Fig. 6:**
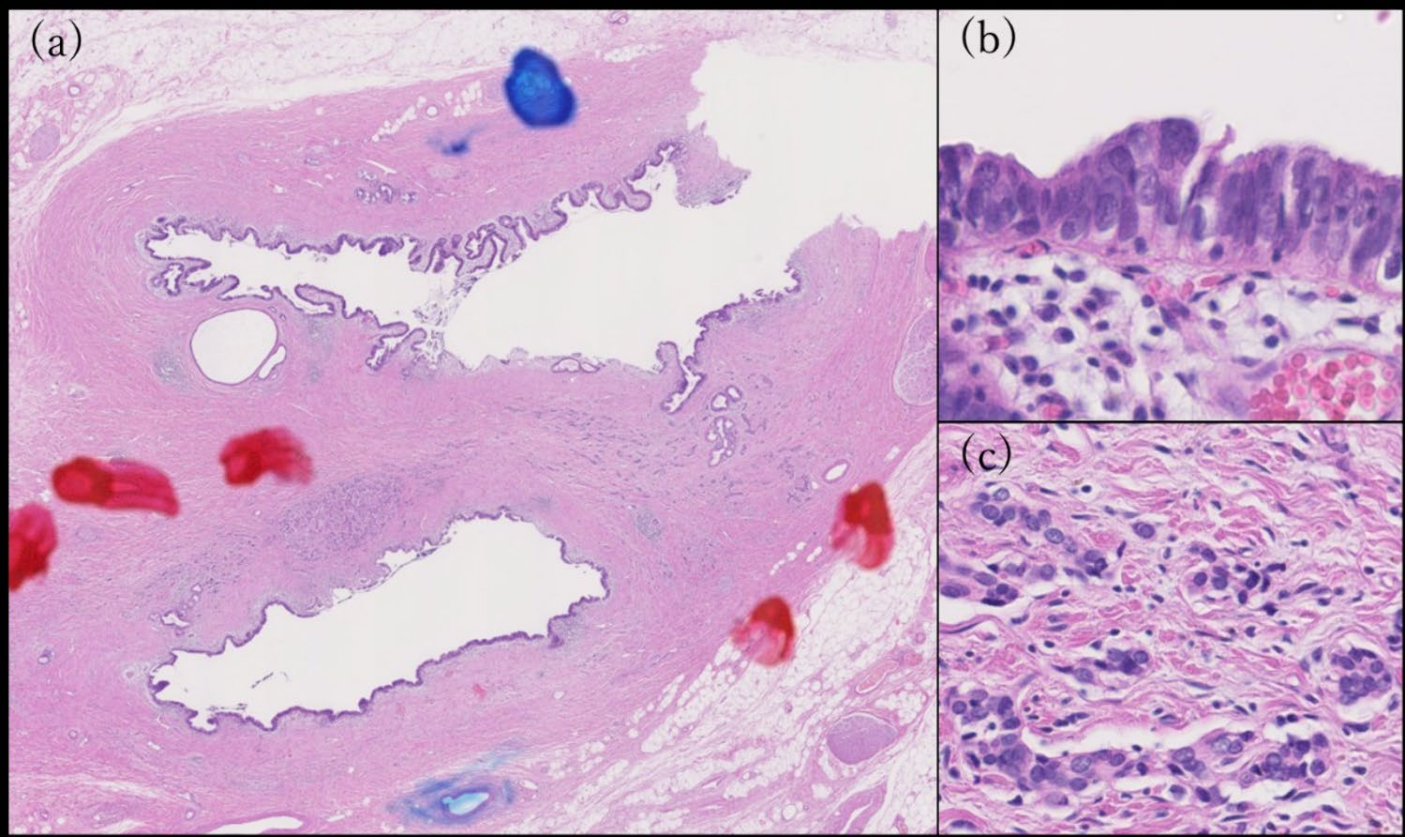
Hematoxylin and eosin staining of bile and cystic ducts **(a)**. Adenocarcinoma is seen in the superficial portion **(b)**, and small tumor cells (SCNEC) are seen in the deeper portion **(c)**

After a conference with the surgeons, a pylorus-preserving pancreatoduodenectomy was performed 3 months after the diagnosis. Postoperative pathology revealed ADC in the mucosal epithelium and scattered SCNEC in the submucosa of the bile duct (Fig. 6).

Because most of the tumors disappeared due to chemotherapy, there was no evidence of a transition between ADC and SCNEC, and the respective occupancy percentages of either component were difficult to assess. Metastasis was found in excised lymph nodes, consisting only of NEC. After six courses of CBDCA + ETP as adjuvant chemotherapy, the treatment was terminated at the patient's request. Thereafter, regular follow-ups were conducted, and recurrence-free survival for 67 months after surgery was achieved.

## Discussion

According to the World Health Organization Classification 2019, NENs are classified as highly differentiated neuroendocrine tumors (NET), poorly differentiated NEC, or MiNENs [2]. It has been reported that primary NEN in the bile duct accounts for approximately 0.22–1.8% of primary NEN in the pancreas and gastrointestinal tract, and little is known about how to diagnose or optimally treat MiNEN of the bile duct [3–5].

The characteristic imaging findings of biliary MiNENs are unclear because of their rarity and heterogeneity. In our case, the lesion presented as a nodular infiltrating type, and a more abundant blood flow signal was observed in the deeper part of the tumor than on the mucosal side on EUS. Compared with the postoperative pathology specimen, the difference in the blood flow signal may suggest two different tumor components with different regional characteristics. The nodular morphology, invasive nature of the tumor, and submucosal mass may reflect the deep proliferation of the NEC component, which arose from the ADC component. Hong et al. reviewed 11 cases of primary extrahepatic bile duct NEN (1 NET, 7 NEC, and 3 mixed adenocarcinoma neuroendocrine carcinoma [MANEC]) and reported that the tumors presented as nodular or intraductal growing-type lesions [8]. Kaino et al. suggested that MiNEN is an admixture of exocrine and endocrine components and that its contrast pattern may be heterogeneous [9]. Therefore, in tumors with lesions with different blood flow characteristics, close examination for MiNEN may be important; however, it is difficult to differentiate MiNEN from cholangiocarcinoma based on imaging alone.

According to a systematic review, the accuracy of the preoperative endoscopic diagnosis was 24.1% in 67 patients who underwent surgical resection for biliary MiNEN [11]. In most cases, the ADC component is located on the mucosal side, and the NEC component is located deeper. It is difficult to detect deeply located NEC components with the commonly performed transpapillary examination, which may be the reason for its low accuracy [10, 11]. Furthermore, the lack of characteristic imaging findings of biliary MiNENs may lead to the diagnosis of cholangiocarcinoma once the ADC component is detected, thus interrupting efforts to detect the NEC component. In MiNEN, NEC is often the cancer component that triggers vascular invasion, liver metastasis, and lymph node metastasis [10, 12], suggesting that histological examination of lymph nodes and liver metastases is likely to detect NEC components. In our case, only the ADC component was detected by transpapillary examination; however, the NEC component was detected by EUS-FNA of the primary tumor and enlarged lymph nodes. For cases in which MiNEN is suspected, it may be necessary to consider not only the transpapillary approach but also the performance of EUS-FNA and the pathological examination of metastases. According to the WHO classification, more than 30% of both components are required to diagnose MiNEN [5, 6], so it is difficult to diagnose MiNEN from biopsy specimens alone. In this case, tumor shrinkage was achieved due to the effects of neoadjuvant chemotherapy, so unfortunately it was not possible to know the exact proportions of both. Therefore, this case cannot be strictly diagnosed as MiNEN. However, there are many similarities between the present case and previous reports. In addition, as a result of comparing the resected specimen and preoperative images, adenocarcinoma was discovered in the same section where the NEC component was present, and it was observed as a single tumor in the image findings before chemotherapy. Therefore, rather than assuming that adenocarcinoma and NEC were present at the same time in the same site, it is reasonable to assume that the tumor contained both components.

Owing to the difficulty of preoperative diagnosis and the rarity of the disease, there is insufficient consensus on treatment for MiNENs with NEC components. In general, treatment for NEC is recommended.

Surgery is the treatment of choice for localized digestive NEC, but relapse is frequent and associated with a poor prognosis. For localized NEC, the presence of regional lymph node metastases and the primary tumor site are considered important prognostic factors [13]. The NCCN guidelines state that treatment options vary depending on the location of the disease, and list neoadjuvant chemotherapy, radiation therapy, chemoradiotherapy, and postoperative adjuvant chemotherapy with or without RT as treatments for resectable NEC [14]. This case was a MiNEN that occurred in the distal bile duct, with lymph node metastasis. The surgical method was pancreaticoduodenectomy, which was highly invasive, and it was suggested that R0 resection might not be possible due to tumor invasion, so we decided to start preoperative chemotherapy. Chemotherapy for NEC recommends combination therapy of cisplatin (CDDP) and ETP, and as an alternative therapy is recommended to use CBDCA instead of CDDP [1, 13]. We chose treatment with CBDCA because the patient was relatively elderly and we wanted to reduce the occurrence of adverse events. There are few reports of neoadjuvant chemotherapy for primary NEC or MiNEN of the bile duct. Terashima et al. reported a case of primary bile duct NEC in which small cell carcinoma was found in biopsy under ERCP and preoperative chemotherapy was performed. CT scan after 2 courses of preoperative chemotherapy (CDDP + ETP) showed no significant change in the size of the tumor, and surgery was performed 2 months after the start of chemotherapy. No postoperative chemotherapy was performed, and CT scan 5 months after surgery showed multiple liver metastases and recurrence [15].

The majority of patients with stage II–III digestive NEC who underwent resection develop recurrence, suggesting that adjuvant chemotherapy may be helpful [13].

A large cohort study of 1861 patients with 519 patients with digestive NEC reported that postoperative chemotherapy was associated with improved survival [16]. Therefore, adjuvant chemotherapy with 4 to 6 cycles of platinum plus ETP may be considered after definitive surgery for local gastrointestinal NEC [13].

Treatment strategies for MiNEN of the bile duct are quite different from those for cholangiocarcinoma. To obtain an accurate preoperative diagnosis, it is important to suspect MiNEN based on atypical tumor images and to make an aggressive histological diagnosis. We believe that patients with MiNEN of the bile duct may benefit from accurate preoperative diagnosis. Neoadjuvant chemotherapy and postoperative adjuvant chemotherapy may be effective treatment options for MiNEN of the bile duct. However, many issues need to be resolved, including the duration of neoadjuvant chemotherapy administration, surveillance intervals, and the timing of planned resection. Therefore, it is desirable to establish a treatment system based on the accumulation and analysis of more cases in the future.
